# Low frequency of local findings in periprosthetic hip infection caused by low-virulent bacteria compared to periprosthetic knee infection

**DOI:** 10.1038/s41598-021-91139-w

**Published:** 2021-06-03

**Authors:** Takashi Imagama, Kazushige Seki, Toshihiro Seki, Yuta Matsuki, Kazuhiro Yamazaki, Takashi Sakai

**Affiliations:** Department of Orthopaedic Surgery, Yamaguchi University Graduate School of Medicine, 1-1-1, Minamikogushi, Ube, 7558505 Japan

**Keywords:** Risk factors, Bacterial pathogenesis, Infectious-disease diagnostics

## Abstract

Periprosthetic joint infection (PJI) is suspected when local findings such as pain, swelling, hyperthermia, and sinus tract are present. However, the frequency of these findings and the difference between hip and knee are unclear. This study compared the positive rates of local findings in periprosthetic hip infection (PHI) with periprosthetic knee infection (PKI), and aimed to identify potential risk factors associated with the frequency. One hundred one PJI (46 hips and 55 knees) fulfilled the 2018 Musculoskeletal infection society criteria were analysed retrospectively to assess the positive rates of each local finding. Patients were categorized into two groups based on the presence or absence of each local finding, and the influence of two potential risk factors [body mass index (BMI) and C-reactive protein (CRP)] was investigated. Causative bacterial species were divided into high and low-virulent groups, and then culture negative cases were included in low-virulent group. PHI had significantly lower rates of pain, swelling and hyperthermia compared to PKI. Overall, up to one-third of PHI had pain as only symptom. High BMI and low-virulent bacteria were associated with lower frequency of swelling and hyperthermia in PHI. CRP had no impact on positive rates of local findings. PHI was oligosymptomatic in a significant percentage of cases. This is particularly important in obese patients and infection by low-virulent bacteria.

## Introduction

Periprosthetic joint infection (PJI) after arthroplasty has been reported to be incidence of 0.2–2.18%^1–3^. In PJI, bacteria gradually form biofilms on the implants that makes them refractory to medical treatment^4^, and implant removal is often necessary. Consequently, two-stage reimplantation involving implant removal is often selected for PJI in cases of delayed diagnosis. In recent year, favorable clinical results of two stage reimplantation for PJI has been reported^5^. However, this has the marked disadvantage of requiring multiple operations, resulting in long-term hospitalization^6^. On the other hand, Löwik et al.^7^ reported that PJI can be cured with debridement only without implant removal, if treated within a week from the onset of infection. As shown here, early diagnosis and treatment are very important to improve the treatment outcomes for PJI.

Bacterial culture of synovial fluid is extremely important in the diagnosis of PJI before surgery. If the causative bacteria are identified, it is very useful in formulating treatment strategies quickly. As the other tests for diagnosis of PJI, synovial fluid leucocyte count^8^, neutrophil percentage^8^, and C-reactive protein^9^ have high sensitivity and specificity (89.5%/91.3%, 89.7%/86.6%, and 85%/95%, respectively). These tests have been included in the 2018 Musculoskeletal Infection Society (MSIS) minor criteria^10^, and their results are very helpful for definitive diagnosis of PJI. However, one of the problems for bacterial culture is that the detection generally needs for several days. This is disadvantage for earlier debridement. In order to improve this problem, it is crucial to suspect PJI earlier and progress the examinations for definitive diagnosis of PJI.

Although joint infections are generally suspected with local findings such as pain, swelling, hyperthermia, and occasionally sinus tract at the initial examination^11,12^, the sensitivity of swelling and hyperthermia was reported to be relatively low (61.5% and 46.2% respectively) in overall PJI^13^. And then, the local findings such as swelling and hyperthermia, and so on excluding sinus tract are not included in the 2018 MSIS criteria for PJI^10^. However, these are the results of overall PJI, and the detail for frequency of local findings is hardly reported compared hip with knee. Additionally, there are no studies whether the virulence of pathogens affects the positive rate of local findings. Knowing the frequency of local findings in each joint helps to improve diagnostic accuracy and definite early diagnosis.

In recent years, some studies described that the pathophysiology in PJI were differ depending on causative bacterial species. PJI with low-virulence bacteria likely resulted in false-negative culture results, and in low levels of C-reactive protein (CRP) in the blood and erythrocyte sedimentation rate (ESR)^14^. Furthermore, it has been reported that CRP and ESR levels can be even lower in culture-negative cases^15^. As described above, causative bacterial species may influence the frequency of local findings in PJI. However, due to a lack of studies for the relationship between frequencies of local findings and virulence of bacteria, the topic remains unclear.

PJI are classified as four subgroups (positive intraoperative cultures, early postoperative infection, acute hematogenous infection, and late chronic infection) depending on the onset and clinical presentation of infection, and the period from surgery^16^. Among them, acute hematogenous infection is reported to be easily accompanied by local findings compared to late chronic infection. Thus, the classification is very important for identifying the frequency of local findings in PJI.

We hypothesized that the positive rates of pain, swelling, hyperthermia, and sinus tract in periprosthetic hip infection (PHI) were relatively lower than in periprosthetic knee infection (PKI). Additionally, we investigated the relationship between the frequency of local findings and three potential risk factors for their absence in PHI. First, body mass index (BMI) was selected as an indicator of soft tissue thickness around the joint. Second, CRP was included as a biochemical marker of joint inflammation severity. Finally, the virulence of causative bacterial species was considered as a risk factor.

In this study, we aimed to determine the different frequency of local findings compared PHI with PKI. Additionally, BMI, CRP, and virulence of causative bacterial species which may affect the frequency of local findings were investigated as risk factors.

## Methods

### Participants and study design

We retrospectively analysed the medical records of 108 patients diagnosed with either PHI or PKI who had been treated by orthopaedic surgeons in our hospital, between April 2008 and July 2020. Patients were excluded if no data on local findings were recorded at the initial examination or if multiple joints were affected. Patients with underlying diseases or receiving medication that may affect CRP levels were also excluded. Finally, 101 joints in 99 patients (46 hips in 46 patients and 55 knees in 53 patients) were included for analysis. The subjects were classified as subgroups (positive intraoperative cultures, early postoperative infection, acute hematogenous infection, and late chronic infection) depending on the onset and clinical presentation of infection, and the period from surgery according to the classification of PJI in previous study^16^. All patients were of Asian origin and met the 2018 MSIS criteria for PJI^10^.

### Evaluation and analysis of local findings

In our hospital, basically minimum two orthopaedic surgeons have assessed the presence or absence for pain, swelling, hyperthermia, and sinus tract compared with contralateral joint during the review period. When opinions were divided, we discussed the case to determine the final assessment. The local findings including pain, swelling, hyperthermia, and sinus tract around the affected joints at the initial examination were retrospectively recorded. Finally, the frequency of each local finding was compared PHI with PKI.

### Measurement of risk factors for the absence of local findings

Patients were categorized into two groups based on the presence or absence of each local finding. BMI and CRP levels were then compared between the two groups in each local finding. Additionally, patients with PHI or PKI were respectively categorized into two groups based on the causative organism: one for low-virulent bacteria (LVB) or culture-negative infections, and the other for high-virulent bacteria (HVB). And then, the frequency of each local finding was compared between HVB and LVB. Both preoperative synovial fluid and intraoperative tissue cultures were performed in all cases. Patients with bacteria detected either preoperative synovial fluid or intraoperative tissue were considered positive. On the other hand, patients who did not detect any bacteria in either test, and met the 2018 MSIS criteria for PJI^10^ were considered culture-negative infection. The virulence of causative bacteria was defined according to a previous study^17^. LVB were included coagulase-negative *Staphylococci*, *Cutibacterium* spp., *Enterococci*, and other bacteria of the normal skin microbioma. The HVB group was included *Staphylococcus aureus*, *Streptococcus* spp., *Enterobacteriaceae*, and nonfermenting gram-negative bacilli. In this study, patients with culture-negative infections were also included in the LVB group because low-virulent pathogens frequently show false-negative culture result in PJI^17^.

### Statistical analysis

In comparison of baseline characteristics of the patients between PHI and PKI, gender, classification of deep periprosthetic infection, culture, and virulence of bacteria were statistically evaluated using Fisher’s exact test. Age, CRP, and BMI were analysed by Mann–Whitney *U* test. Comparisons of local findings between PHI and PKI, or LVB and HVB groups were made using Fisher’s exact test. Differences in BMI or CRP between presence and absence of each local finding were analysed using Mann–Whitney *U* test. Values are shown as medians (interquartile range). Graph Pad Prism software version 8.0 (GraphPad, San Diego, CA, USA) was used for analyses; p-values < 0.05 were regarded as statistically significant.

### Ethics approval

This study was performed in line with the principles of the Declaration of Helsinki. Approval was granted by the Ethics Committee and Institutional Review Board of Yamaguchi University (H2019-003-2).

### Informed consent

Informed consent was obtained from all individual participants included in the study.

## Results

### Patient characteristics

Patients with PKI were significantly older than those with PHI (71 [67–76] years vs. 75 [73–78] years; p < 0.05). Forty-six PHI included 35 total hip arthroplasties and 11 bipolar hip arthroplasties, while 55 PKI included 53 total knee arthroplasties and 2 unicompartmental knee arthroplasties. Bacterial cultures yielded positive results in 89.1% (41/46) of PHI and 80.0% (44/55) of PKI, respectively. LVB accounted for 45.7% (21/46) of PHI and 40.0% (22/55) of PKI. Regarding PHI specifically, *Staphylococcus aureus* was isolated in 20 joints, *Streptococcus* spp. in 3 joints, coagulase-negative *staphylococci* in 13 joints, *Enterococci* in 2 joints, and *Enterobacteriaceae*, nonfermenting gram-negative bacilli, and *Cutibacterium* spp. in one joint. In PKI, *Staphylococcus aureus* was isolated in 29 joints, *Streptococcus* spp. in 3 joints, coagulase-negative *staphylococci* in 10 joints, and *Enterococci* and *Enterobacteriaceae* in 1 joint, respectively. BMI in PHI was significantly lower than that of the knee (p < 0.01), whereas CRP levels was not statistically different between PHI and PKI. The subjects in PHI were classified as 0 joint in positive intraoperative cultures, 4 joints in early postoperative infection, 41 joints in acute hematogenous infection, and 1 joint in late chronic infection. Each classification in PKI was 0, 6, 48, and 1 joint, respectively. There was no significant difference between both groups in classification of deep periprosthetic infection (Table 1).

**Table 1:** Baseline characteristics of the patients.

	PHI	PKI	p value
Number (joint)	46	55	
Gender, male:female	17:29	21:34	> 0.999
Age (year), median (interquartile range)	71 (67–76)	75 (73–78)	0.029
Surgical procedure (joint)	THA:BHA 35:11	TKA:UKA 53:2	NA
**Classification of deep periprosthetic infection (joint)**			
Positive intraoperative cultures	0	0	NA
Early postoperative infection	4	6	0.711
Acute hematogenous infection	41	48	0.774
Late chronic infection	1	1	0.898
CRP (mg/dL), median (interquartile range)	7.5 (5.1–9.8)	7.6 (2.4–18.4)	0.504
BMI, median (interquartile range)	22.0 (20.6–23.3)	23.8 (22.1–24.8)	0.006
Culture positive:negative	41:5	44:11	0.277
High:low virulent bacteria	25:21	33:22	0.687

*PHI* periprosthetic hip infection, *PKI* periprosthetic knee infection, *THA* total hip arthroplasty, *BHA* bipolar hip arthroplasty, *TKA* total knee arthroplasty, *UKA* unicompartmental knee arthroplasty, *NA* not available, *CRP* C-reactive protein, *BMI* body mass index.

### Comparison of local findings

**Figure 1:**
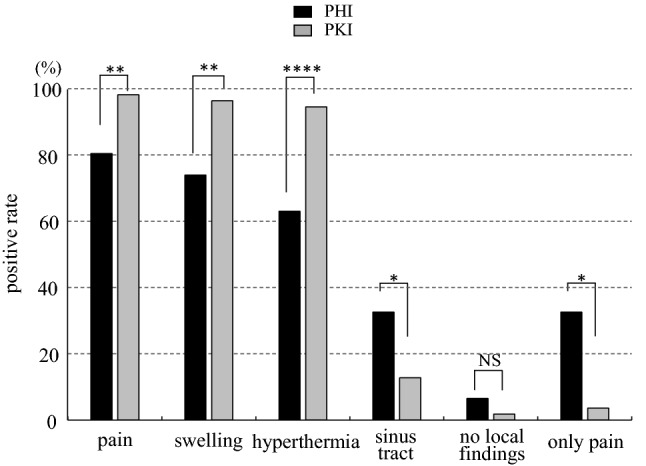
Positive rates of local findings in periprosthetic hip infection (PHI) and periprosthetic knee infection (PKI). The positive rates of pain, swelling, and hyperthermia in PHI were significantly lower than PKI. Only sinus tract in PHI was significantly higher than PKI. The frequency of no local findings was not significant difference between the groups. PHI showed a significantly higher frequency in the presence of pain as the only symptom compared to PKI. ****p < 0.0001, **p < 0.01, *p < 0.05, *NS* no significant difference.

Pain was appearing in 80.4% of the hip and 98.2% of the knee cases, and the positive rate of PHI was significantly lower than PKI (p < 0.01). The positive rates of swelling and hyperthermia were significantly lower in PHI than PKI (73.9% vs 96.4%, p < 0.01; 63.0% vs 94.5%, p < 0.0001; respectively). On the contrary, the appearance of sinus tract was significantly less frequent in PKI than PHI (12.7% vs. 32.6%, respectively; p < 0.05). Additionally, 6.5% (3/46) of PHI and 1.8% (1/55) of PKI presented no local findings (no significant difference). The patients who had only pain were 32.6% (15/46) of PHI and 3.6% (2/55) of PKI. PHI showed to be significantly higher rate than PKI (p < 0.05) (Fig. 1).

### BMI, CRP, and causative bacterial species as risk factors for the absence of local findings in PHI

**Figure 2:**
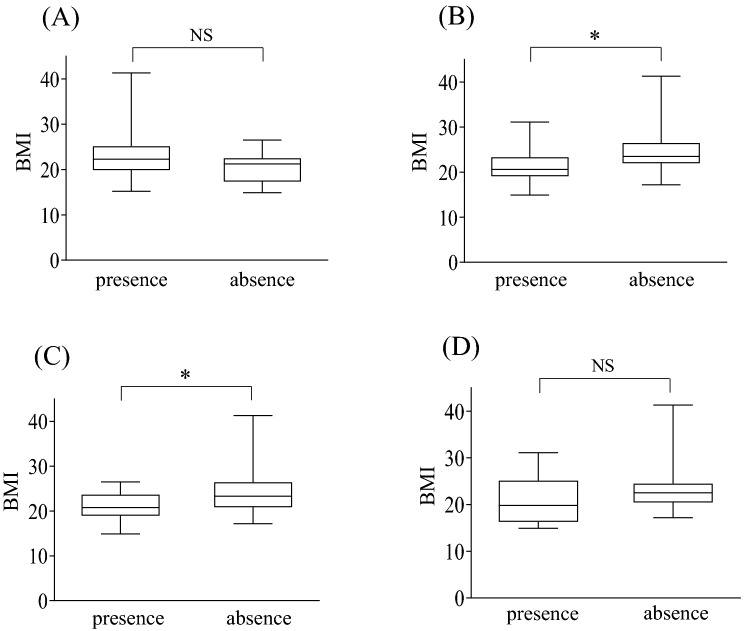
Comparison between body mass index (BMI) of patients with presence and absence of local findings in periprosthetic hip infection (PHI). The patients with swelling or hyperthermia had significantly lower BMI than those without them in PHI. Pain (**A**), swelling (**B**), hyperthermia (**C**), and sinus tract (**D**) *p < 0.05, *NS* no significant difference.

**Figure 3:**
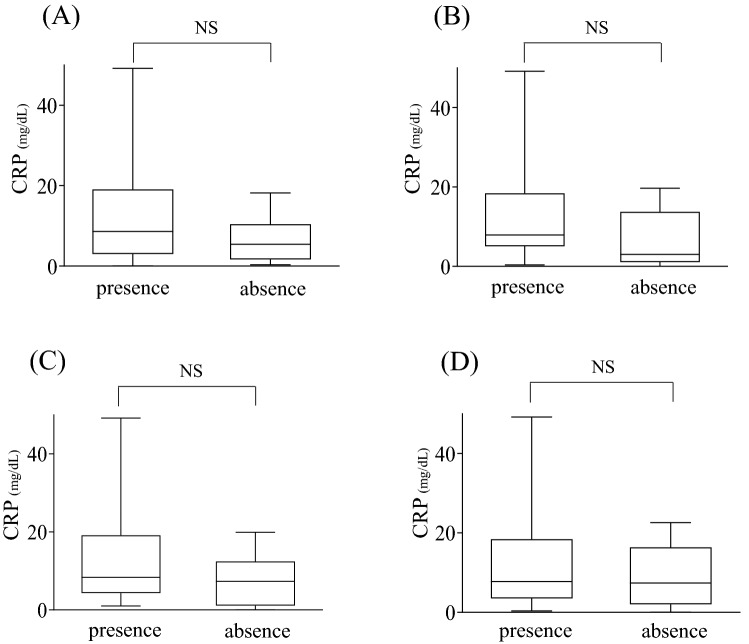
Comparison between C-reactive protein (CRP) levels of patients with presence and absence of local findings in periprosthetic hip infection (PHI). The significant difference of CRP was not shown between two groups in PHI. Pain (**A**), swelling (**B**), hyperthermia (**C**), and sinus tract (**D**). *NS* no significant difference.

Patients with swelling and hyperthermia had significantly lower BMIs than those without such local findings in PHI (Fig. 2). CRP levels were not significantly different between the patients with and without local findings (Fig. 3). In PKI the number of negative cases in pain, swelling, hyperthermia, and positive cases in sinus tract was too small, thus the comparison was not conducted in the present study.

### Influence of virulence of causative bacteria for the frequency of local findings

**Figure 4:**
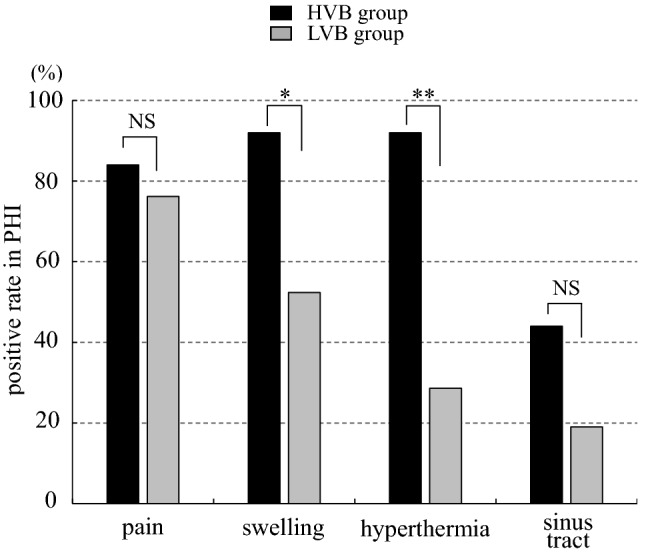
Frequency of local findings according to virulence of causative bacteria in periprosthetic hip infection (PHI). The positive rates of swelling and hyperthermia were significantly lower in low-virulent bacteria (LVB) group than high-virulent bacteria (HVB) group. **p < 0.01, *p < 0.05, *NS* no significant difference.

**Figure 5:**
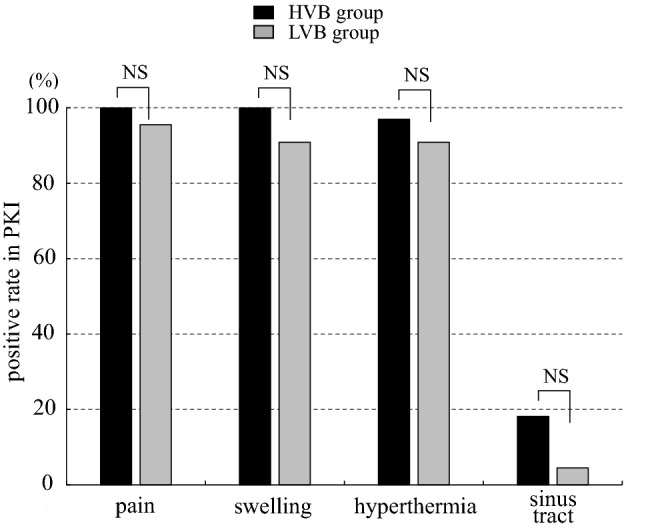
Frequency of local findings according to virulence of causative bacteria in periprosthetic knee infection (PKI). The significant difference of the frequency of each local finding was not shown between high and low-virulent bacteria in PKI. *LVB* low-virulent bacteria, *HVB* high-virulent bacteria, *NS* no significant difference.

Regarding the impact of the virulence of causative bacteria for local findings in PHI, the positive rates of pain, swelling, hyperthermia, and sinus tract were 84.0%, 92.0%, 92.0%, and 44.0% in HVB group, and 76.2%, 52.4%, 28.6%, and 19.0% in the LVB group, respectively. Swelling and hyperthermia were significantly less frequent in LVB group than in HVB group (p < 0.05 and < 0.01, respectively). The positive rates of pain and sinus tract were not significantly different between the groups (Fig. 4). In contrast, in PKI the frequency of pain, swelling, hyperthermia, and sinus tract were 100%, 100%, 97.0%, and 18.2% in the HVB group, and 95.5%, 90.9%, 90.9%, and 4.5% in the LVB group, respectively. The positive rates of all local findings were not significant difference between the groups (Fig. 5).

## Discussion

In the present study, pain, swelling, and hyperthermia were significantly less frequent, and sinus tract was contrarily more frequent in PHI compared with PKI. Interestingly, about 33% of PHI occurred without local findings but with pain as the only symptom. Moreover, a higher BMI was significantly associated with the absence of swelling and hyperthermia in PHI. And then infection by low-virulent bacteria had a significantly low frequency of swelling and hyperthermia in PHI. In contrast, in PKI, the frequency of all local findings was not affected for virulence of causative bacteria. This is the first report to compare the frequency of local findings such as pain, swelling, hyperthermia, and sinus tract in patients with PHI and PKI, and to indicate the risk factors influenced the frequency.

In PJI, bacteria form biofilms that make them resistant to antibiotics^4^. In this setting, medical treatment is usually ineffective and implant removal is essential for a definitive cure^6^. However, some studies have shown that implant preservation is feasible if debridement is performed early in the course of the infection. A study reported successful outcomes when debridement was performed within 1 week after symptom onset^7^. Moreover, Brandt et al.^18^ described that in cases of *S. aureus* infection, debridement with prosthesis preservation should be performed within only two days of symptom onset. Thus, the interval from symptom onset to surgery is an important factor for implant retention in PJI.

Usually, local findings are the first clue to musculoskeletal infection, and the presence of them leads to make early diagnosis. In particular, swelling and hyperthermia have high diagnostic value in native joint septic arthritis^19^ and superficial incisional surgical site infection^20^. Our study revealed that the frequencies of pain, swelling, and hyperthermia were lower in PHI than PKI. Although pain is present in the majority infections, regardless of the site, pain alone is not useful for early diagnosis of joint infection because it can be present in a variety of conditions such as aseptic loosening of PJI, rheumatoid arthritis and crystal arthritis. Thus, our results indicated that the absence of these local findings would not help to rule out PHI, and each local finding would have a different diagnostic value between hip and knee.

Due to its high specificity, sinus tract is a major criterion for PJI diagnosis according to the guidelines of the MSIS^10^. In this study, although the presence of sinus tract was not frequent in PHI and PKI, the positive rate was higher in hip than knee unlike the other local findings. In this regard, the low frequency of swelling and hyperthermia in PHI may have allowed the infection to progress unnoticed, leading to the appearance of this complication. However, this mechanism is not clear, and further investigation is needed.

We hypothesized that BMI, CRP levels, and virulence of causative bacteria affect the frequency of local findings. BMI was significantly higher in patients with PHI who presented no swelling or hyperthermia. The reason for this result is considered that the incidence of these local findings is affected by the thickness of soft tissue from the skin to the joint. Because hip joint is deeply located compared to other joints including knee, the presence of local findings would be lower than PKI.

Regarding the severity of joint inflammation, we found that CRP levels was not related to the frequency of any local findings in PHI. Thus, CRP was considered not to be risk factor for the low frequency of local findings in PHI. This result may suggest that the thickness of soft tissue in PHI affects local findings, regardless of the degree of inflammation. This indicates that even the patients with high CRP levels do not always have local findings. This knowledge would be useful to prevent misdiagnosis.

As compared with the virulence of causative bacteria, low virulent bacteria were associated with lower rates of swelling and hyperthermia than high-virulent bacteria in PHI. In contrast, the frequency of local findings was not significantly different between low and high virulent bacteria in PKI. These results are the first report to refer to the relationship between local findings and virulence of causative bacterial species. Several previous studies described the differences in characteristics depending on the causative bacterial species in PJI. Morgenstern et al.^17^ stated that synovial fluid culture in PJI was inferior for detection of low-virulent organisms. A study by Alijanipour et al.^21^ demonstrated intraoperative purulence was less detected in PJI caused by low-virulent bacteria compared to high virulent bacteria. Moreover, Kheir et al.^15^ reported that laboratory values such as CRP, erythrocyte sedimentation rate, synovial white blood cell count, and polymorphonuclear percentage in patients with low-virulent organisms or culture negative PJI often were not elevated. These show that the pathophysiology of infection would differ depending on the causative bacterial species. Similar to these reports, based on the result that the frequency of local findings in PHI was lower in LVB group in this study, we should be aware of the low frequency for local findings associated with low-virulent bacteria. Conversely, the frequency of local findings in PKI was not different dependent on causative bacterial species. Since the positive rates of local findings were basically high in PKI, it may have been difficult to detect a difference depending on causative bacterial species.

This study has several limitations. First, due to the low incidence of PJI, a small number of patients was included. Further studies with larger numbers of subjects are required to confirm our results, however, this research is significant as a pilot study. Second, we did not perform any analysis to investigate the validation of local findings for diagnosis of PJI. Determining the sensitivity, specificity, positive predictive value, and negative predictive value of local findings alone or in combination would be of great value. Finally, although local findings were subjectively evaluated by two surgeons, we should consider the quantitative evaluation of each local finding. For example, a thermography or non-contact thermometer should be used for assessing hyperthermia in future research.

In conclusion, our results showed that the frequencies of local findings excluded sinus tract in PHI were significantly lower than those in PKI. The absence of local findings was unreliable for excluding PHI compared to PKI. In addition, there was oligosymptomatic in a significant percentage of patients in PHI, especially in the setting of a high BMI and/or infection due to low-virulent bacteria. Our results would help to enable an earlier diagnosis of PHI. We consider that it is always important to maintain a high level of suspicion to avoid delays in treatment that may result in removing prosthesis, even if there are no local findings in PHI.
